# Dengue in Bali: Clinical characteristics and genetic diversity of circulating dengue viruses

**DOI:** 10.1371/journal.pntd.0005483

**Published:** 2017-05-22

**Authors:** Dewi Megawati, Sri Masyeni, Benediktus Yohan, Asri Lestarini, Rahma F. Hayati, Febrina Meutiawati, Ketut Suryana, Tangking Widarsa, Dewa G. Budiyasa, Ngurah Budiyasa, Khin S. A. Myint, R. Tedjo Sasmono

**Affiliations:** 1 Faculty of Medicine and Health Sciences, Warmadewa University, Jl. Terompong No. 24 Denpasar, Bali, Indonesia; 2 Eijkman Institute for Molecular Biology, Jl. Diponegoro No. 69, Jakarta, Indonesia; 3 Wangaya General Hospital. Jl. Kartini No 133 Denpasar, Bali, Indonesia; 4 Sanjiwani General Hospital. Jl. Ciung Wenara No 2 Gianyar, Bali, Indonesia; Duke-NUS GMS, SINGAPORE

## Abstract

A high number of dengue cases are reported annually in Bali. Despite the endemicity, limited data on dengue is available for Bali localities. Molecular surveillance study was conducted to explore the clinical and virological characteristics of dengue patients in urban Denpasar and rural Gianyar areas in Bali during the peak season in 2015. A total of 205 adult dengue-suspected patients were recruited in a prospective cross-sectional study. Demographic and clinical information were obtained, and dengue screening was performed using NS1 and IgM/IgG ELISAs. Viral RNA was subsequently extracted from patients’ sera for serotyping using conventional RT-PCR and Simplexa Dengue real-time RT-PCR, followed by genotyping with sequencing method. We confirmed 161 patients as having dengue by NS1 and RT-PCR. Among 154 samples successfully serotyped, the DENV-3 was predominant, followed by DENV-1, DENV-2, and DENV-4. Serotype predominance was different between Denpasar and Gianyar. Genotyping results classify DENV-1 isolates into Genotype I and DENV-2 as Cosmopolitan Genotype. The classification grouped isolates into Genotype I and II for DENV-3 and DENV-4, respectively. Clinical parameters showed no relationship between infecting serotypes and severity. We observed the genetic diversity of circulating DENV isolates and their relatedness with historical data and importation to other countries. Our data highlights the role of this tourist destination as a potential source of dengue transmission in the region.

## Author summary

Dengue is the most significant mosquito-borne viral disease affecting humans. Up to one third of the world population is at risk of dengue virus (DENV) infection, transmitted through the bite of *Aedes* mosquitoes. Bali, a well-known international tourist destination, is regularly ravaged by dengue disease. This disease impacts the health of both local people and visitors thus imposing a heavy economic burden. Bali has a constant flow of travelers and labors that contribute to the spread of DENV infection. Detailed characterization of DENV from Bali is limited; most reports are from travel-acquired cases. Here, we study dengue clinical and virological aspects in local Balinese people. We presented the clinical spectrum of the disease and the virological characteristics, observing the circulation of genetically diverse endemic virus strains including strains which are closely related to imported viruses in neighboring countries. The circulation of a lineage of DENV-2 proposed to cause outbreak in the past is also identified. Our study provides data on the genetic of circulating DENV in Bali which are useful for further applications, such as to monitor the virus transmission and outbreak investigation in the region.

## Introduction

Dengue is the most important arthropod-borne disease affecting humans with high incidence in tropical and subtropical countries. It is estimated that 390 million infections occur annually and over 70% of the world population is at risk of being infected by dengue viruses (DENVs) [1]. Dengue can manifest complex clinical features; infection with any of the four antigenically distinct DENVs may lead to a range of clinical manifestations, which vary in severity from classic dengue fever (DF) to a more severe and fatal dengue hemorrhagic fever (DHF) and dengue shock syndrome (DSS) [2]. DENV, a member of the *Flaviviridae* family, consists of a 10.7 kb single-stranded positive-sense RNA genome encoding three structural (C, prM/M, E) and seven non-structural (NS1,NS2A, NS2B, NS3, NS4A, NS4B, NS5) proteins [3]. The substantial genetic diversity of DENV is shown by the presence of various genotypes within the four DENV serotypes (DENV-1, -2, -3, and -4) [4,5].

Bali is a well-known international tourist destination located in the tropical country of Indonesia and is regularly affected by dengue disease. This disease affects the health of both local people and visitors imposing a heavy economic burden [1,6]. It has been reported that Bali has a constant flow of labor and travelers that contribute to the spread of DENV infection [7]. Major outbreaks occurred in 2010 and 2015 with 12,574 (including 35 fatalities) and 10,704 (28 fatalities) reported dengue cases, respectively (*Dinas Kesehatan Provinsi Bali*/Bali Provincial Health Office).

Previous reports documented the hyperendemic transmission of all four DENV serotypes in Bali during 2010, and the circulating DENV included the dominant local strains which had circulated for several years, as well as strains more recently introduced into Bali [8]. This transmission created substantial DENV diversity and serve as a hub for dengue transmission and mixing in Bali [8]. Most of the reports of dengue in travelers returning from Indonesia have implicated Bali as the source of importation [8–10]. All four DENV serotypes were detected from travelers entering Western Australia between 2010–2012, mostly from Bali [8]. It has also been reported that highest proportion (24.6%) of imported dengue cases of travelers in Queensland, Australia originated from Indonesia [11]. Despite the year-round transmission and large cyclical outbreaks observed in travelers from Bali, no substantial data of clinical and genetic features of dengue in local Balinese people are currently available.

To obtain comprehensive data on dengue disease in local Balinese, we have conducted molecular surveillance to characterize clinical aspects and genetic diversity of the DENVs circulating in Bali during the high dengue season in 2015. We report here the demographic, clinical, hematological, and virological characteristics of dengue in adult hospitalized patients in two geographically different regions, namely the urban Denpasar municipality and rural Gianyar regency. The data will be beneficial for the control of this disease in Bali and also highlights the potential of this island as the source of imported dengue cases to other parts of the world.

## Materials and methods

### Ethics statements

The study protocol was approved by the Medical Research Ethics Committee of Faculty of Medicine Udayana University/Sanglah Central General Hospital, Bali Indonesia with approval No. 122/UN.14.2/Litbang/2015.

### Study site, patient recruitment, and sample collection

The cross-sectional prospective study was conducted during the period of March to May 2015. Samples were collected from Wangaya (WGY) and Sanjiwani (SJN) General Hospitals which are located in Denpasar municipality and Gianyar regency, respectively. Denpasar and Gianyar were recorded as the regions with the highest dengue incidents in Bali in 2014 [12]. Inpatients (above 14 years) presenting at the adult wards with fever >38°C accompanied by at least one sign of dengue such as malaise, arthralgia, rash, retro-orbital pain, DHF or DSS were enrolled in the study after providing written informed consents. We excluded patients with history of chronic illnesses, such as chronic liver disease, diabetes mellitus, chronic kidney disease, chronic lung disease, human immunodeficiency syndrome, and cardiac disease. Sera were collected during the acute phase (within the first five days of illness) and before discharge from the hospital. Each patient’s demographic, clinical and hematological data as well as disease severity according to the WHO-SEARO 2011 guidelines were recorded [13].

### DENV NS1 antigen detection and serological tests

The preliminary screening for DENV in patients’ sera was performed using the SD Bioline NS1 rapid test (Alere, Australia), according to the manufacturer’s instructions. Serological tests were performed using Panbio Dengue Duo IgM/IgG Capture ELISA (Alere). The serological test results were used to determine the primary/secondary infection status of patients, as described previously [14]. Confirmation using Panbio IgG indirect ELISA (Alere) was also performed, in which the presence of previous IgG antibody to DENV would indicate secondary infection.

### RNA extraction and RT-PCR detection and serotyping

DENV viral RNA was extracted from acute serum samples using QIAmp Viral RNA Mini kit (Qiagen, Hilden, Germany) according to the manufacturer’s instructions. The DENV detection and serotyping was carried out according to the two step protocol as previously described by Lanciotti *et al*.[15] with some modification [16]. The more sensitive Simplexa Dengue (Focus Diagnostics, Cypress, USA) qRT-PCR was used as a confirmatory assay to simultaneously detect and serotype DENV as previously described [17].

### DENV envelope gene sequencing

Samples were genotyped based on Envelope (E) gene. DENV RNA was reverse-transcribed into cDNA using Superscript III reverse transcriptase (Invitrogen-Life Technologies) and then PCR-amplified using *Pfu* Turbo Polymerase (Stratagene-Agilent Technologies)[18]. PCR products were purified from 0.8% agarose gel using the QIAquick gel extraction kit (Qiagen) and cycle sequencing reactions were performed using six overlapping primers for each serotype from both strands with Big Dye Dideoxy Terminator sequencing kits v3.1 (Applied Biosystems-Life Technologies), as described previously [18]. Purified DNA was subjected to capillary sequencing performed on 3130xl Genetic Analyzer (Applied Biosystems). Sequence reads were assembled using SeqScape v.2.5 software (Applied Biosystems) with manual inspections employed whenever ambiguities were present. Sequence contigs were generated and used in subsequent analyses.

### DENV genotype analyses

Genotyping of DENV was based on classifications by Goncalvez et al. [19], Twiddy et al.[20], Lanciotti et al. [21], and Lanciotti et al. [22] for DENV-1, -2, -3 and -4, respectively. Envelope gene sequences of Bali isolates together with other representative sequences downloaded from GenBank were aligned using MUSCLE in MEGA 6.0 software (http://www.megasoftware.net/). The initial dataset was prepared using BEAUti graphical interface and the tip of each isolate calibrated using the year of isolation as calibration point. Phylogenetic tree was inferred based on selection of statistical model for likelihood calculation optimized for Maximum Likelihood (ML) tree using jModelTest v.2.1.4 [23]. Phylogenetic reconstruction and evolutionary rate analysis were performed using Bayesian Markov chain Monte Carlo (MCMC) method as implemented in BEAST v.1.8.2 [24]. Runs were performed using General Time Reversible (GTR) model with four gamma parameters (GTR + Γ4) and relaxed uncorrelated lognormal molecular clock using the initial estimated evolutionary rate of 7.6 x 10^−4^ substitutions per site per year, as previously described [25]. The tree prior was set as coalescent bayesian skyline prior, to facilitate the fewest demographic assumptions. One hundred million chains were run and sampled for every 1000^th^ iteration, with 10% burn-in employed. The convergence of parameters was analyzed using Tracer v.1.5.0 to ensure adequate Effective Sampling Size (ESS) for all parameters. Maximum clade credibility (MCC) tree was created using TreeAnnotator v.1.8.2 and visualized in FigTree v.1.4.0.

### Statistical analysis

Pearson’s Chi-square or Fisher’s Exact tests were used to compare univariate categorical data. Parametric One-way ANOVA or non-parametric Kruskal-Wallis tests were used to compare groups of laboratory test results within DENV serotypes. The regression analyses were performed using modified Poisson regression or the ordinal logistic regression with adjustment of clinically relevant potential covariates, i.e. age, gender, recruitment site, infection status, and fever at day of presentation. A probability value of *p* < 0.05 was considered statistically significant. All statistical analyses were performed using Stata version 12 (StataCorp, TX).

### Accession numbers

The complete E gene sequences of 28 DENV isolates were deposited in GenBank repository and granted accession numbers KY006129 to KY006156 (Supplementary S1 Table).

## Results

### Patient characteristics

A total of 205 dengue-suspected cases were recruited from two hospitals in Bali Province. This comprises of 99 (48.3%) and 106 (51.7%) samples from Wangaya General Hospital, Denpasar, and Sanjiwani General Hospital, Gianyar respectively. The majority of the patients were recruited on day 4 or 5 of illness. The median age of the patients was 29 years, with a total range of 14–80 years. Equal proportions of male and female were observed with no significant differences in terms of gender and age (Table 1). Two patients did not meet the inclusion criteria and were excluded from analysis.

**Table 1:** Demographic characteristics and diagnosis of study participants related to the infecting DENV serotypes.

Variables	DENV-1	DENV-2	DENV-3	DENV-4	Mix	*p* value
	N = 43 (%)	N = 26 (%)	N = 74 (%)	N = 6 (%)	N = 5 (%)	
*Sex*						
Male	19 (44.2)	10 (38.5)	32 (43.2)	3 (50.0)	4 (80.0)	0.549
Female	24 (55.8)	16 (61.5)	42 (56.8)	3 (50.0)	1 (20.0)	
*Age*						
Adult (14–50 yr.)	36 (83.7)	26 (100.0)	66 (89.2)	5 (83.3)	4 (80.0)	0.243
Elderly (>50 yr.)	7 (16.3)	0 (0.0)	8 (10.8)	1 (16.7)	1 (20.0)	
*Study site*						
Denpasar	14 (32.6)	12 (46.2)	41 (55.4)	6 (100.0)	1 (20.0)	**0.008**
Gianyar	29 (67.4)	14 (53.8)	33 (44.6)	0 (0.0)	4 (80.0)	
*NS1 antigen detection*						
NS1 positive	41 (95.3)	22 (84.6)	73 (98.6)	6 (100.0)	5 (100.0)	0.054
NS1 negative	2 (4.7)	4 (15.4)	1 (1.4)	0 (0.0)	0 (0.0)	
*Infection status*						
Primary	8 (18.6)	3 (11.5)	13 (17.6)	1 (16.7)	0 (0.0)	0.797
Secondary	35 (81.4)	23 (88.5)	61 (82.4)	5 (83.3)	5 (100.0)	

The NS1 antigen detection confirmed 154 (75.9%) cases of dengue infection. Further confirmatory tests using RT-PCR were performed on NS1-negative samples and resulted in seven additional dengue-confirmed samples, for a total number of 161 (79.3%). Serology testing revealed that the majority of the cases were of secondary infection (83.8%) while the remaining 16.2% were of primary infection (Table 1).

### DENV serotype distribution

We successfully serotyped 154 out of 161 (95.6%) dengue-confirmed samples. All four serotypes were found to circulate in Bali during the study period. DENV-3 was predominant (48%), followed by DENV-1 (28%), DENV-2 (17%), and DENV-4 (4%). Five samples (3%) were detected as mixed infection of two different serotypes (DENV-1 and -2 and DENV-1 and -3). The predominance of DENV-3 was evident in both study sites. However, there were differences in the proportions of the other serotypes (Table 1). While six DENV-4 were found in Denpasar, the presence of DENV-4 in Gianyar was only detected as one case of mixed infection with DENV-3. The distribution of dengue serotypes between two regions was significantly different (*p =* 0.008) based on univariate statistical analysis. Other variables i.e. sex, age, and infection status were not significantly different between serotypes (Table 1).

### Clinical manifestations, hematological parameters, and disease severity

Analysis of dengue clinical manifestations and hematological parameters were performed excluding the DENV-4 and mix infection cases due to the small sample size. Following the adjustment based on age, gender, infection status, site of study, and day of fever by the time of presentation, we observed no correlation between clinical manifestations and the infecting serotypes. The statistical analysis indicated that DENV-2-infected patients were likely to have 0.23 times the risk of loss of appetite compared to infection with other serotypes (95% CI = 0.07–0.75) (Table 2). The only hematological parameter difference between dengue serotypes was higher diastolic blood pressure in patients infected with DENV-1 (*p =* 0.042) (Table 3). In terms of disease severity, 75 patients (52.4%) were DF with the remaining 68 (47.6%) patients classified as DHF, where four patients were identified as DHF grade III (DSS) with hematemesis. Similarly, we did not find any correlation between disease severity and the infecting serotype (Table 4).

**Table 2:** Clinical parameters observed in dengue patients in relation to the DENV serotypes*.

Variables	DENV-1 (N = 43)		DENV-2 (N = 26)		DENV-3 (N = 74)	
	N (%)	RR (95% CI)	N (%)	RR (95% CI)	N (%)	RR (95% CI)
Malaise	41 (95.3%)	1.45 (0.28–7.64)	24 (92.3%)	2.44 (0.40–14.90)	68 (91.9%)	0.79 (0.29–2.15)
Nausea	36 (83.7%)	0.69 (0.25–1.93)	23 (88.5%)	2.19 (0.46–10.35)	66 (89.2%)	1.03 (0.41–2.54)
Loss of appetite	39 (90.7%)	1.72 (0.51–5.79)	18 (69.2%)	**0.23 (0.07–0.75)**	62 (83.8%)	1.26 (0.59–2.72)
Headache	35 (81.4%)	1.14 (0.43–3.02)	18 (69.2%)	0.79 (0.27–2.29)	53 (71.6%)	1.01 (0.54–1.90)
Myalgia	30 (69.8%)	0.97 (0.41–2.26)	16 (61.5%)	1.57 (0.50–4.97)	43 (58.1%)	0.93 (0.50–1.73)
Vomiting	22 (51.2%)	0.78 (0.38–1.59)	14 (53.8%)	0.79 (0.33–1.93)	44 (59.5%)	1.19 (0.70–2.02)
Arthralgia	26 (60.5%)	0.89 (0.38–2.09)	11 (42.3%)	0.42 (0.14–1.32)	39 (52.7%)	1.27 (0.69–2.34)
Abdominal pain	16 (37.2%)	1.13 (0.55–2.32)	11 (42.3%)	2.16 (0.79–5.95)	16 (21.6%)	0.70 (0.37–1.32)
Retro-orbital pain	12 (27.9%)	1.00 (0.46–2.17)	8 (30.8%)	2.75 (0.85–8.84)	16 (21.6%)	0.77 (0.39–1.51)
Bleeding	13 (30.2%)	1.57 (0.78–3.13)	3 (11.5%)	0.59 (0.17–2.05)	14 (18.9%)	0.81 (0.44–1.50)

*Analysis was performed excluding DENV-4 and mix infection groups due to small sample size. Relative risk (RR) and confidence intervals (CI) were calculated based on modified Poisson regression adjusted for age, gender, primary/secondary infection, recruitment site, and fever day at presentation.

**Table 3:** Vital signs and hematological parameters in relation to the DENV serotypes*.

Variables	DENV-1 (N = 43)		DENV-2 (N = 26)		DENV-3 (N = 74)	
	Median	*p* value	Median	*p* value	Median	*p* value
	(Range)		(Range)		(Range)	
Temperature (^o^C)	37.7	0.530	37.7	0.582	38	0.333
	(35.7–40)		(36–39.3)		(36–40)	
Systolic blood pressure (mmHg)	110	0.196	110	0.277	110	0.135
	(90–140)		(90–120)		(80–170)	
Diastolic blood pressure (mmHg)	80	**0.042**	70	0.807	70	0.107
	(60–90)		(60–80)		(50–110)	
Pulse/minute	82	0.121	84	0.899	84	0.426
	(66–100)		(78–110)		(72–108)	
Platelet count nadir (10^9^/L)	44	0.234	36	0.764	29.5	0.374
	(9–101)		(10–105)		(6–130)	
White blood cells count (10^9^/L)	2.2	0.817	2.0	0.321	2.1	0.599
	(0.6–3.94)		(0.8–3.95)		(0.6–6.4)	
Hematocrit level (%)	43.2	0.886	41.9	0.892	41.9	0.920
	(34–81.8)		(29.4–51.1)		(33.5–79.1)	
Hemoglobin (g/dL)	15.2	0.360	14.3	0.175	14.8	0.752
	(12.1–19.5)		(10.8–16.8)		(11.6–19.6)	

*Analysis was performed excluding DENV-4 and Mix groups due to small sample size.

**Table 4:** Relationship between DENV serotypes and disease severity*.

Variables	N	DF			DHF		
		N (%)	RR (95% Cl)	aRR (95% Cl)	N (%)	RR (95% Cl)	aRR (95% Cl)
DENV-1	43	26 (60.5)	0.95 (0.41–2.19)	1.24 (0.74–2.06)	17 (39.5)	1.09 (0.37–3.23)	0.77 (0.43–1.39)
DENV-2	26	12 (46.2)	0.73 (0.29–1.84)	0.93 (0.48–1.80)	14 (53.8)	1.48 (0.49–4.50)	1.07 (0.57–1.99)
DENV-3	74	37 (50)	0.79 (0.66–1.24)	1.00 (NA)	37 (50)	1.10 (0.80–1.51)	1.00 (NA)

*Relative risk (RR) and confidence intervals (CI) were calculated based on modified Poisson regression and adjusted (aRR) for age, gender, primary/secondary infection, recruitment site, and fever day at presentation. NA, not applicable.

### DENV genotypes distribution

**Fig 1:**
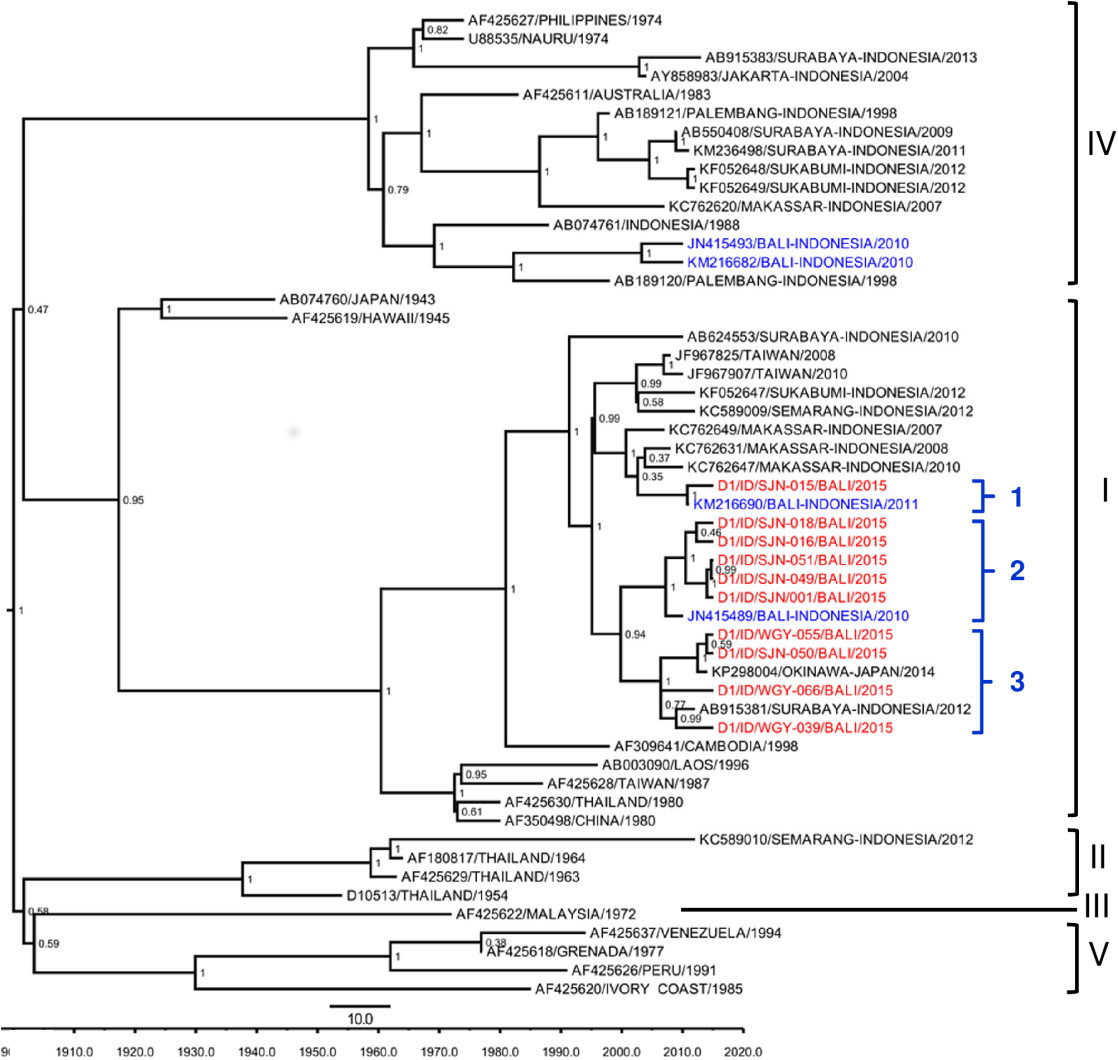
Phylogenetic tree and genotype classification of DENV-1 isolates from Bali generated using Bayesian inference method based on E gene sequences. The Bali 2015 isolates (red font) were grouped into Genotype I based on classification by Goncalvez et al. [19], together with previous strains from Bali (blue font). Arabic numbers denote lineage grouping. The posterior probabilities of the clades were indicated as numbers in the node labels.

To determine the genotypes of DENV within each serotype in Bali in 2015, we performed genotyping based on E gene sequences. We successfully obtained complete sequences of E gene from 28 patients’ sera. Of 43 DENV-1 isolates, 10 (23.3%) were successfully PCR-amplified for their E genes. Phylogenetic analysis revealed that all 10 isolates were grouped into Genotype I based on Goncalvez [19] classification (Fig 1). Although grouped in a single genotype, the 10 Bali DENV-1 isolates were further differentiated into six lineages. It was also notable that the Genotype IV isolates that were present in Bali in 2010 (Fig 1) were not found in this study.

**Fig 2:**
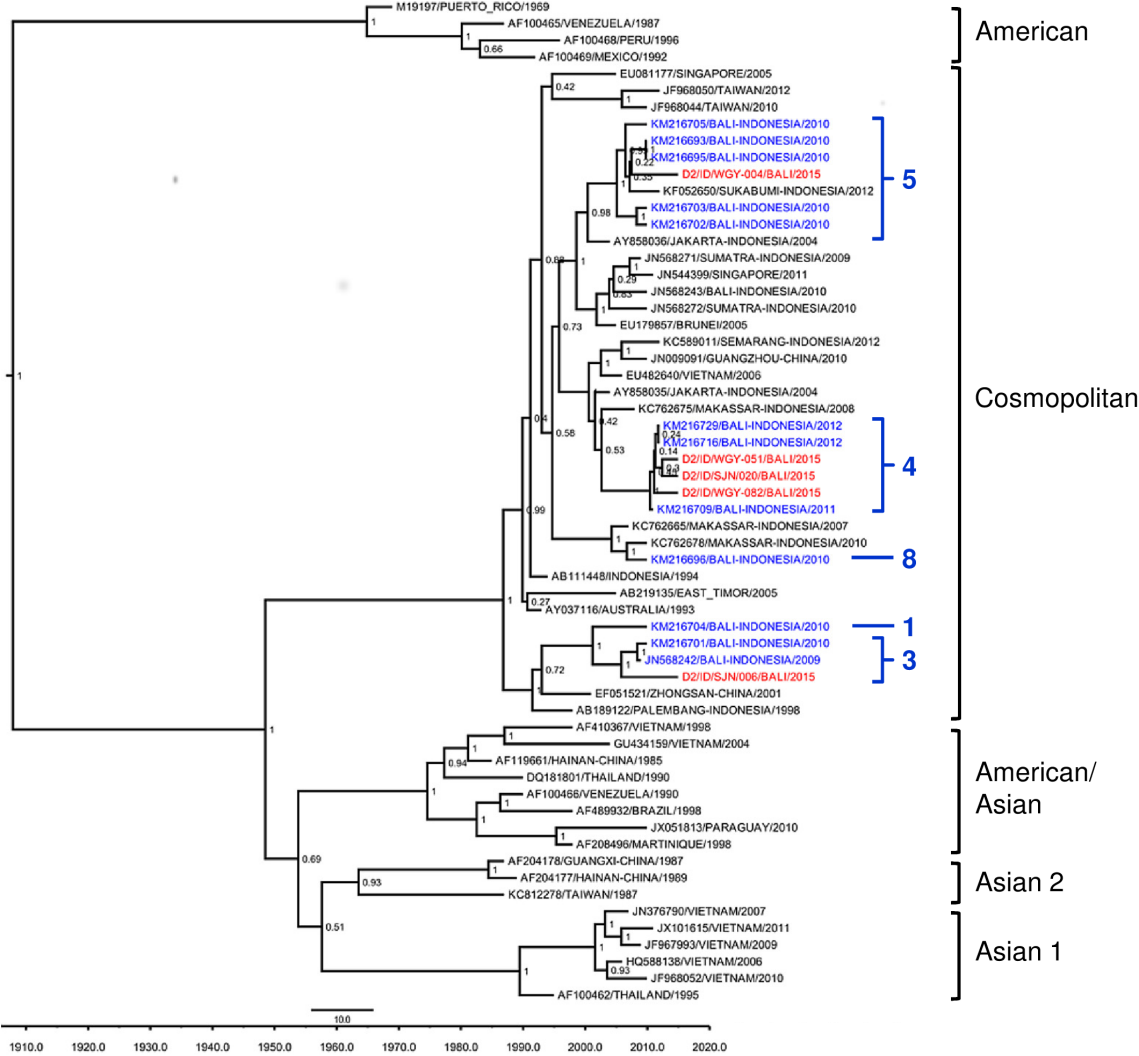
Phylogenetic tree and genotypes classification of DENV-2 isolates from Bali generated using Bayesian inference method based on E gene sequences. The Bali 2015 isolates (red font) were grouped into the Cosmopolitan Genotype based on classification by Twiddy et al. [20], together with previous strains from Bali (blue font). Further lineages were classified according to Ernst et al. [8] (in Arabic numbers). The posterior probabilities of the clades were indicated as numbers in the node labels.

For DENV-2, five isolates were successfully genotyped out of 26 isolates. Phylogenetic analysis revealed that all of these five isolates belonged to Cosmopolitan genotype according to Twiddy [20] classification (Fig 2). These five viruses of three distinct lineages formed a monophyletic clade with previous Bali isolates circulating in 2010 [8].

**Fig 3:**
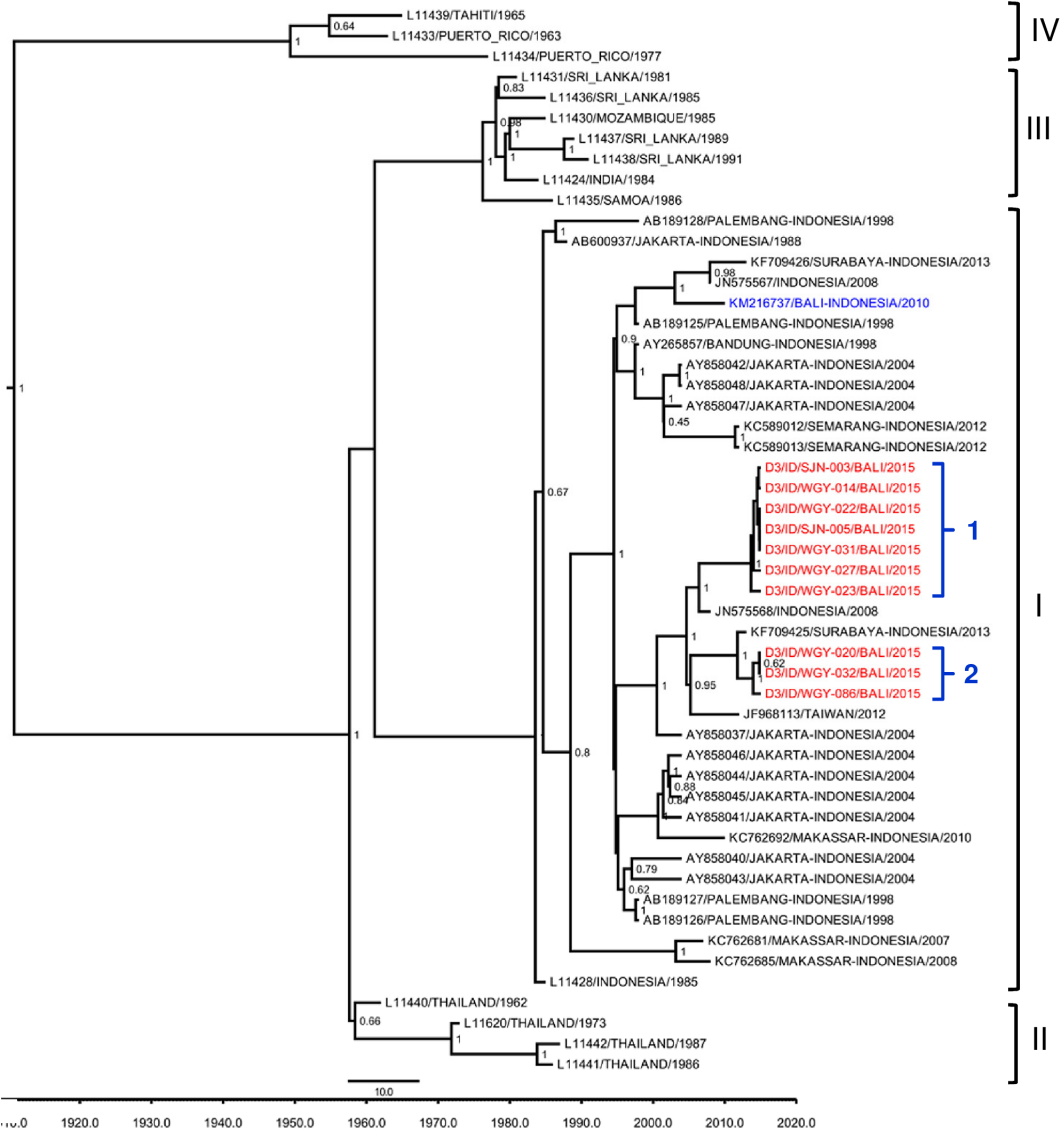
Phylogenetic tree and genotype classification of DENV-3 isolates from Bali generated using Bayesian inference method based on E gene sequences. The Bali 2015 isolates (red font) were grouped into Genotype I based on classification by Lanciotti et al. [21], together with previous strains from Bali (blue font). Arabic numbers denote lineage grouping. The posterior probabilities of the clades were indicated as numbers in the node labels.

We also genotyped 10 isolates of DENV-3 which were grouped as Genotype I based on Lanciotti [21] classification and were further differentiated into two major lineages (Fig 3). Both lineages appeared to have different ancestral origin from other DENV-3 isolated in Bali in 2010.

**Fig 4:**
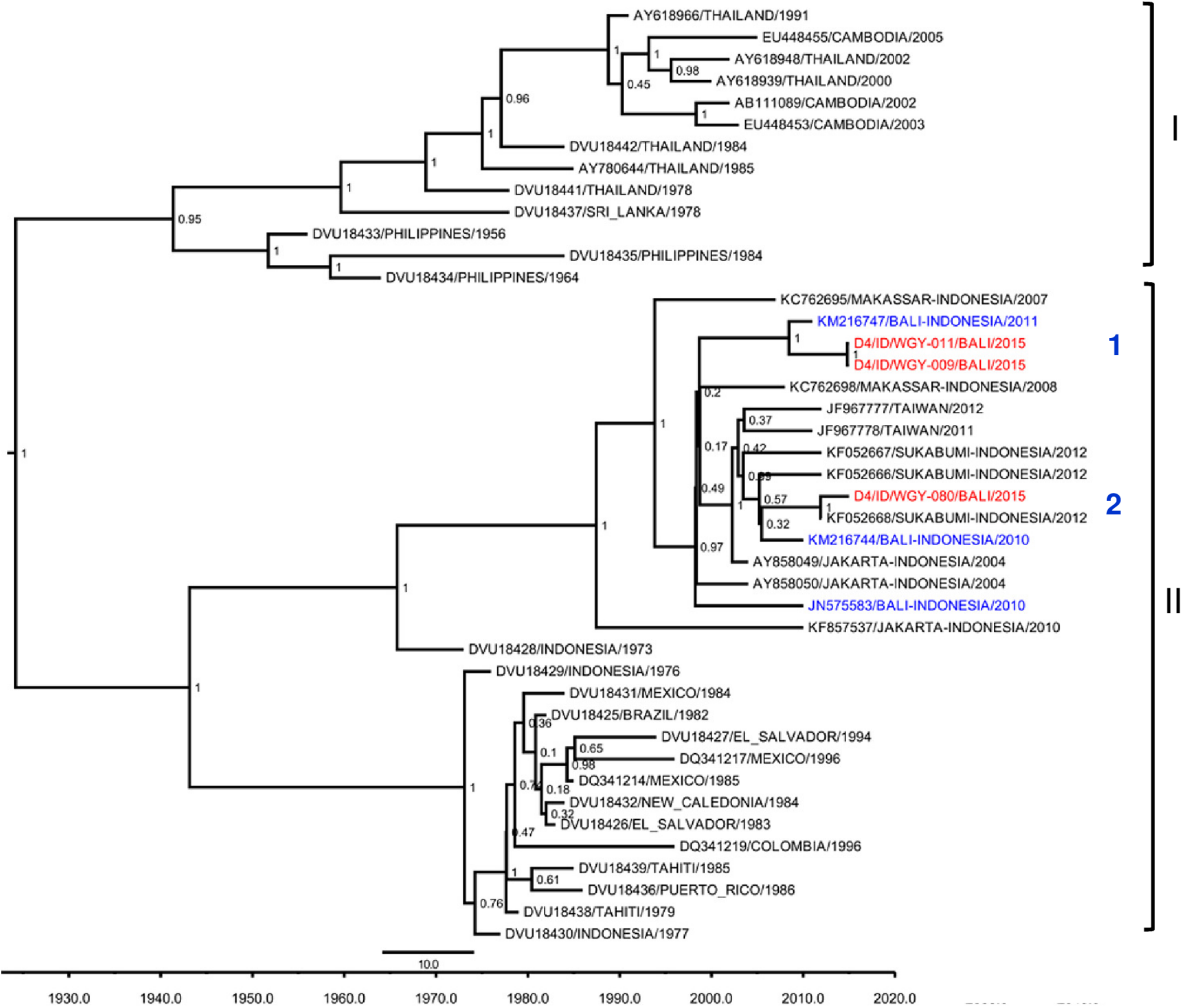
Phylogenetic tree and genotypes classification of DENV-4 isolates from Bali generated using Bayesian inference method based on E gene sequences. The Bali 2015 isolates (red font) were grouped into Genotype II based on classification by Lanciotti et al. [22], together with previous strains from Bali (blue font). Arabic numbers denote lineage grouping. The posterior probabilities of the clades were indicated as numbers in the node labels.

For DENV-4, from six isolates serotyped, three isolates were successfully sequenced and genotyped. Following Lanciotti classification [22], all three viruses were grouped as Genotype II (Fig 4). Two separate lineages were observed, and the Bali 2015 isolates formed a monophyletic clade with Bali 2010 isolates as well as other Indonesia isolates from Sukabumi, Makassar, and Jakarta. A grouping with DENV isolates imported to Taiwan was also observed.

## Discussion

Bali Province is an island of approximately 5,780 km^2^ in area and located in the tropical climate zone (latitude -8.4095178 and longitude 115.188916). Having one municipality and eight regencies, the province is inhabited by 3,995,281 residents [12]. The data provided by the Provincial Health Office showed fluctuating numbers of dengue cases during the period of 2009 to 2015. We conducted the first virological investigation of dengue in Bali to determine the genomic diversity and its relation to the clinical manifestations.

In this study, we confirmed 79.3% (161/203) of patients as dengue positive, suggesting the considerable burden of dengue in the community. The majority (83.8%) of the confirmed cases were of secondary dengue infection based on serology results. This result was as expected since the recruited patients were adults with more prolonged exposure to dengue infection in the past. This number confirmed the endemicity of dengue in Bali.

All four DENV serotypes were found circulating in Bali in 2015. The most prevalent serotype was DENV-3, followed by DENV-1, DENV-2, and DENV-4. With lack of molecular data for the dengue virus in Bali, no comparison could be made to the current predominant serotypes identified in our study. The predominance of DENV-3 has been reported in Indonesia [26–28]. In our molecular surveillance, we observed the rise of DENV-1 infection as the second most common serotype. Serotype replacement has been described in a number of reports [14,18,29,30]. However, further surveillance is needed to monitor the dynamics of DENV in Bali in order to confirm this phenomenon. Although the two study sites (the Denpasar municipality and Gianyar Regency) are only about 28 km apart, statistical analysis showed that the distribution of the serotypes between the two regions was significantly different (*p* = 0.008). This discrepancy may have resulted from the different demographic profile of the sites. Sanjiwani Hospital is a referral hospital for the Gianyar regency in eastern part of Bali which mostly consisted of rural areas with less dense population (1345 people per km^2^), while the patients admitted to Wangaya Hospital, Denpasar mostly resided in densely populated areas around Denpasar (population density 6891 people per km^2^) [12] which is also a major domestic and international tourist destination. The different population density may account for the different transmission profile of particular serotypes, as has been observed in Viet Nam [31].

On the clinical aspect of dengue in Bali, a higher number of patients with DF was observed rather than DHF (Table 1), even though the majority of the patients were adults with secondary infection. It was reported that secondary infection is a risk factor for increased severity [32]. However, we did not observe a correlation between the higher number of secondary infection and increased severity as in other studies [33].

The effect of serotypes on clinical manifestations of dengue fever in adults has been reported [34,35]. A previous study in adults in Singapore reported that joint pain and red eyes were associated with DENV-2 and DENV-1, respectively [36]. Within all the clinical variables observed, we found the loss of appetite as the only parameter with significant correlation with DENV-2 (Table 2). Similarly, the only vital signs and hematological parameters that significantly correlated with different serotypes was higher diastolic blood pressure observed in DENV-1-infected patients (Table 3) which was not reported in the published literature. Interestingly, we did not find a correlation between thrombocytopenia and infecting serotype, as observed in other studies conducted in adults [36,37]. In addition, there was no correlation between other clinical parameters and the disease severity (Supplementary S2 Table).

In terms of virological aspects, the DENV E gene sequences for 28 representative isolates were generated. This provides DENV genetic data from Bali that will be useful for various applications such as molecular epidemiology studies and outbreak investigations. Phylogenetic analysis revealed that all of the 10 DENV -1 isolates were grouped into Genotype I (Fig 1). The isolates were closely related to Bali strains from imported cases to Australia in 2010 [9] and 2011 [8] and Japan [10] and strains from other cities in Indonesia i.e. Makassar [18] and Surabaya [29]. The genetic diversity of DENV-1 in Bali is extensive as shown by the presence of multiple lineages within Genotype I group (Fig 1). In this study, we did not find the other DENV-1 genotype (Genotype IV) known to circulate in Bali in 2010 [38] and other cities in Indonesia as well as in imported cases to other countries. The absence of Genotype IV in Bali together with genotype replacement is similar to that seen with the Jambi dengue outbreak [30], and other cities of Indonesia [18,29]. Altogether, our data suggest the ongoing replacement of DENV-1 Genotype IV by Genotype I in Bali.

The Cosmopolitan DENV-2 isolates were further clustered into different lineages and closely related to the imported dengue cases to Australia during 2009–2011 [8]. Several lineages have been reported within the Cosmopolitan genotype of DENV-2 from imported cases to Australia, mostly from Bali [8]. Following the lineage numbering, Bali 2015 isolates were clustered into lineages 3, 4, and 5 (Fig 2). Of the five Bali DENV-2 isolates sequenced, three isolates belong to this lineage. The lineage 4 has been described to have emerged during a major outbreak in Bali in 2011–2012 [8]. Isolates from Denpasar and Gianyar were grouped into this lineage, reflecting the spread of this lineage in Bali. Our study confirms the active circulation of this particular lineage in Bali and the potential active transmission and exportation to other regions.

Contrary to the data reported by Ernst et al [8] which showed the predominance of DENV-2 in Australian travelers visiting Bali in 2010, DENV-3 was the predominant serotype in Bali in 2015, indicating the shifting of serotypes in Bali. DENV transmission is dynamic and serotypes have been known to show cyclical predominant pattern which may correlate with herd immunity [39]. Genetically, ten isolates of Bali DENV-3 were grouped into Genotype I (Fig 3) which is the common DENV-3 genotype found in Indonesia [14,18,30,40,41]. The Bali 2015 isolates were grouped together with isolates of imported cases to Australia and Taiwan and those from Surabaya and Jakarta [9,41–43] The Bali 2015 DENV-3 isolates apparently were not as divergent as DENV-1 and -2, in which only two major lineages were observed. This might suggest that the DENV-3 that caused outbreaks in Bali were the local/endemic strains that have been circulating in the region for decades and not those introduced from outside of Indonesia. DENV-4 was the least prevalent in Bali, in which only six isolates were found. Among these, three isolates were grouped into Genotype II (Fig 4) which is commonly found in Indonesia [14,18,40,41]. The isolates were closely related to imported dengue cases to Australia and isolates from Sukabumi, a city in West Java Province [9,40]. Again, we did not observe any introduced DENV-4 strains in Bali which suggests that the DENV-4 in Bali were the local and endemic strains.

In summary, our study provides the first detailed information on the clinical and virological features of dengue in two areas in Bali with the highest dengue cases in 2015. Our study has some limitations related to potential selection bias based on recruitment criteria as only adults were enrolled, a relatively small number of samples were collected; and the time period of collection was limited. Nevertheless, we confirmed the hyperendemicity of all four DENV serotypes where the circulating DENV included dominant local strains which were in circulation for several years and were related to recent imported dengue cases to other countries. Our study highlights Bali as a place with prominent genetic diversity of DENV and supports previous reports on its role in dengue transmission and mixing. Further studies on active molecular surveillance of DENV should be done in Bali to monitor dengue dynamics.

## Supporting information

S1 Checklist STROBE Checklist. (PDF)

S1 Table Characteristics of sequenced samples. (PDF)

S2 Table Clinical parameters of dengue patients in relation to the disease severity. (PDF)

## References

[1] Bhatt S, Gething PW, Brady OJ, Messina JP, Farlow AW, Moyes CL, et al The global distribution and burden of dengue. Nature. 2013;496: 504–507. doi: 10.1038/nature12060 23563266 10.1038/nature12060 PMC3651993

[2] Martina BEE, Koraka P, Osterhaus ADME. Dengue virus pathogenesis: an integrated view. Clin Microbiol Rev. 2009;22: 564–581. doi: 10.1128/CMR.00035-09 19822889 10.1128/CMR.00035-09 PMC2772360

[3] Guzman MG, Halstead SB, Artsob H, Buchy P, Farrar J, Gubler DJ, et al Dengue: a continuing global threat. Nat Rev Microbiol. 2010;8: S7–16. doi: 10.1038/nrmicro2460 21079655 10.1038/nrmicro2460 PMC4333201

[4] Holmes EC, Burch SS. The causes and consequences of genetic variation in dengue virus. Trends Microbiol. 2000;8: 74–7. 10664600 10.1016/s0966-842x(99)01669-8

[5] Holmes EC, Twiddy SS. The origin, emergence and evolutionary genetics of dengue virus. Infect Genet Evol. 2003;3: 19–28. 12797969 10.1016/s1567-1348(03)00004-2

[6] Shepard DS, Undurraga EA, Halasa YA, Stanaway JD. The global economic burden of dengue: a systematic analysis. Lancet Infect Dis. 2016;16: 935–941. doi: 10.1016/S1473-3099(16)00146-8 27091092 10.1016/S1473-3099(16)00146-8

[7] Yoshikawa MJ, Kusriastuti R. Surge of dengue virus infection and chikungunya Fever in bali in 2010: the burden of mosquito-borne infectious diseases in a tourist destination. Trop Med Health. 2013;41: 67–78. doi: 10.2149/tmh.2011-05 23874141 10.2149/tmh.2011-05 PMC3705185

[8] Ernst T, McCarthy S, Chidlow G, Luang-Suarkia D, Holmes EC, Smith DW, et al Emergence of a new lineage of dengue virus type 2 identified in travelers entering Western Australia from Indonesia, 2010–2012. PLoS Negl Trop Dis. 2015;9: e0003442 doi: 10.1371/journal.pntd.0003442 25635775 10.1371/journal.pntd.0003442 PMC4311992

[9] Warrilow D, Northill JA, Pyke AT. Sources of dengue viruses imported into Queensland, australia, 2002–2010. Emerging Infect Dis. 2012;18: 1850–1857. doi: 10.3201/eid1811.120014 23092682 10.3201/eid1811.120014 PMC3559152

[10] Saito M, Tamayose M, Miyagi K, Takaragawa H, Tateyama M, Tadano M, et al Serologic and Virologic Studies of an Imported Dengue Case Occurring in 2014 in Okinawa, Japan. Jpn J Infect Dis. 2016;69: 60–65. doi: 10.7883/yoken.JJID.2015.061 26073738 10.7883/yoken.JJID.2015.061

[11] Viennet E, Ritchie SA, Faddy HM, Williams CR, Harley D. Epidemiology of dengue in a high-income country: a case study in Queensland, Australia. Parasit Vectors. 2014;7: 379 doi: 10.1186/1756-3305-7-379 25138897 10.1186/1756-3305-7-379 PMC4261250

[12] Bali Provincial Health Office. Bali Province Health Profile 2015. 2016.

[13] WHO-SEARO. Comprehensive guidelines for prevention and control of dengue and dengue haemorrhagic fever Revised and expanded. New Delhi, India: World Health Organization; 2011.

[14] Fahri S, Yohan B, Trimarsanto H, Sayono S, Hadisaputro S, Dharmana E, et al Molecular surveillance of dengue in Semarang, Indonesia revealed the circulation of an old genotype of dengue virus serotype-1. PLoS Negl Trop Dis. 2013;7: e2354 doi: 10.1371/journal.pntd.0002354 23951374 10.1371/journal.pntd.0002354 PMC3738473

[15] Lanciotti RS, Calisher CH, Gubler DJ, Chang GJ, Vorndam AV. Rapid detection and typing of dengue viruses from clinical samples by using reverse transcriptase-polymerase chain reaction. J Clin Microbiol. 1992;30: 545–551. 1372617 10.1128/jcm.30.3.545-551.1992 PMC265106

[16] Harris E, Roberts TG, Smith L, Selle J, Kramer LD, Valle S, et al Typing of dengue viruses in clinical specimens and mosquitoes by single-tube multiplex reverse transcriptase PCR. J Clin Microbiol. 1998;36: 2634–2639. 9705406 10.1128/jcm.36.9.2634-2639.1998 PMC105176

[17] Sasmono RT, Aryati A, Wardhani P, Yohan B, Trimarsanto H, Fahri S, et al Performance of Simplexa dengue molecular assay compared to conventional and SYBR green RT-PCR for detection of dengue infection in Indonesia. PLoS ONE. 2014;9: e103815 doi: 10.1371/journal.pone.0103815 25102066 10.1371/journal.pone.0103815 PMC4125142

[18] Sasmono RT, Wahid I, Trimarsanto H, Yohan B, Wahyuni S, Hertanto M, et al Genomic analysis and growth characteristic of dengue viruses from Makassar, Indonesia. Infect Genet Evol. 2015 10.1016/j.meegid.2015.03.006 25784569

[19] Goncalvez AP, Escalante AA, Pujol FH, Ludert JE, Tovar D, Salas RA, et al Diversity and evolution of the envelope gene of dengue virus type 1. Virology. 2002;303: 110–119. 12482662 10.1006/viro.2002.1686

[20] Twiddy SS, Farrar JJ, Vinh Chau N, Wills B, Gould EA, Gritsun T, et al Phylogenetic relationships and differential selection pressures among genotypes of dengue-2 virus. Virology. 2002;298: 63–72. 12093174 10.1006/viro.2002.1447

[21] Lanciotti RS, Lewis JG, Gubler DJ, Trent DW. Molecular evolution and epidemiology of dengue-3 viruses. J Gen Virol. 1994;75 (Pt 1): 65–75. 8113741 10.1099/0022-1317-75-1-65

[22] Lanciotti RS, Gubler DJ, Trent DW. Molecular evolution and phylogeny of dengue-4 viruses. J Gen Virol. 1997;78 (Pt 9): 2279–2284. 9292015 10.1099/0022-1317-78-9-2279

[23] Darriba D, Taboada GL, Doallo R, Posada D. jModelTest 2: more models, new heuristics and parallel computing. Nat Methods. 2012;9: 772. 10.1038/nmeth.2109 PMC4594756 22847109

[24] Drummond AJ, Rambaut A. BEAST: Bayesian evolutionary analysis by sampling trees. BMC Evol Biol. 2007;7: 214 doi: 10.1186/1471-2148-7-214 17996036 10.1186/1471-2148-7-214 PMC2247476

[25] Costa RL, Voloch CM, Schrago CG. Comparative evolutionary epidemiology of dengue virus serotypes. Infect Genet Evol. 2012;12: 309–314. doi: 10.1016/j.meegid.2011.12.011 22226705 10.1016/j.meegid.2011.12.011

[26] Setiati TE, Wagenaar JF, de Kruif MD, Mairuhu AT, van Gorp EC, Soemantri A. Changing epidemiology of dengue haemorrhagic fever in Indonesia. Bull WHO. 2006;30: 1–14.

[27] Corwin AL, Larasati RP, Bangs MJ, Wuryadi S, Arjoso S, Sukri N, et al Epidemic dengue transmission in southern Sumatra, Indonesia. Trans R Soc Trop Med Hyg. 2001;95: 257–65. 11490992 10.1016/s0035-9203(01)90229-9

[28] Suwandono A, Kosasih H, Nurhayati, Kusriastuti R, Harun S, Ma’roef C, et al Four dengue virus serotypes found circulating during an outbreak of dengue fever and dengue haemorrhagic fever in Jakarta, Indonesia, during 2004. Trans R Soc Trop Med Hyg. 2006;100: 855–62. doi: 10.1016/j.trstmh.2005.11.010 16507313 10.1016/j.trstmh.2005.11.010

[29] Yamanaka A, Mulyatno KC, Susilowati H, Hendrianto E, Ginting AP, Sary DD, et al Displacement of the predominant dengue virus from type 2 to type 1 with a subsequent genotype shift from IV to I in Surabaya, Indonesia 2008–2010. PLoS ONE. 2011;6: e27322 doi: 10.1371/journal.pone.0027322 22087290 10.1371/journal.pone.0027322 PMC3210158

[30] Haryanto S, Hayati RF, Yohan B, Sijabat L, Sihite IF, Fahri S, et al The molecular and clinical features of dengue during outbreak in Jambi, Indonesia in 2015. Pathog Glob Health. 2016; 1–11. 27215933 10.1080/20477724.2016.1184864 PMC4984957

[31] Raghwani J, Rambaut A, Holmes EC, Hang VT, Hien TT, Farrar J, et al Endemic dengue associated with the co-circulation of multiple viral lineages and localized density-dependent transmission. PLoS Pathog. 2011;7: e1002064 doi: 10.1371/journal.ppat.1002064 21655108 10.1371/journal.ppat.1002064 PMC3107208

[32] Guzman MG, Alvarez M, Halstead SB. Secondary infection as a risk factor for dengue hemorrhagic fever/dengue shock syndrome: an historical perspective and role of antibody-dependent enhancement of infection. Arch Virol. 2013;158: 1445–1459. doi: 10.1007/s00705-013-1645-3 23471635 10.1007/s00705-013-1645-3

[33] Guilarde AO, Turchi MD, Siqueira JB, Feres VCR, Rocha B, Levi JE, et al Dengue and dengue hemorrhagic fever among adults: clinical outcomes related to viremia, serotypes, and antibody response. J Infect Dis. 2008;197: 817–824. doi: 10.1086/528805 18269315 10.1086/528805

[34] Tsai JJ, Chan KS, Chang JS, Chang K, Lin CC, Huang JH, et al Effect of serotypes on clinical manifestations of dengue fever in adults. J Microbiol Immunol Infect. 2009;42: 471–478. 20422131

[35] Soo K-M, Khalid B, Ching S-M, Chee H-Y. Meta-Analysis of Dengue Severity during Infection by Different Dengue Virus Serotypes in Primary and Secondary Infections. PLoS ONE. 2016;11: e0154760 doi: 10.1371/journal.pone.0154760 27213782 10.1371/journal.pone.0154760 PMC4877104

[36] Yung C-F, Lee K-S, Thein T-L, Tan L-K, Gan VC, Wong JGX, et al Dengue serotype-specific differences in clinical manifestation, laboratory parameters and risk of severe disease in adults, Singapore. Am J Trop Med Hyg. 2015;92: 999–1005. doi: 10.4269/ajtmh.14-0628 25825386 10.4269/ajtmh.14-0628 PMC4426593

[37] Fried JR, Gibbons RV, Kalayanarooj S, Thomas SJ, Srikiatkhachorn A, Yoon I-K, et al Serotype-specific differences in the risk of dengue hemorrhagic fever: an analysis of data collected in Bangkok, Thailand from 1994 to 2006. PLoS Negl Trop Dis. 2010;4: e617 doi: 10.1371/journal.pntd.0000617 20209155 10.1371/journal.pntd.0000617 PMC2830471

[38] Aryati A, Trimarsanto H, Yohan B, Wardhani P, Fahri S, Sasmono RT. Performance of commercial dengue NS1 ELISA and molecular analysis of NS1 gene of dengue viruses obtained during surveillance in Indonesia. BMC Infect Dis. 2013;13: 611 doi: 10.1186/1471-2334-13-611 24571329 10.1186/1471-2334-13-611 PMC3905968

[39] Adams B, Holmes EC, Zhang C, Mammen MP, Nimmannitya S, Kalayanarooj S, et al Cross-protective immunity can account for the alternating epidemic pattern of dengue virus serotypes circulating in Bangkok. Proc Natl Acad Sci USA. 2006;103: 14234–14239. doi: 10.1073/pnas.0602768103 16966609 10.1073/pnas.0602768103 PMC1599940

[40] Nusa R, Prasetyowati H, Meutiawati F, Yohan B, Trimarsanto H, Setianingsih TY, et al Molecular surveillance of Dengue in Sukabumi, West Java province, Indonesia. J Infect Dev Ctries. 2014;8: 733–741. doi: 10.3855/jidc.3959 24916872 10.3855/jidc.3959

[41] Ong SH, Yip JT, Chen YL, Liu W, Harun S, Lystiyaningsih E, et al Periodic re-emergence of endemic strains with strong epidemic potential-a proposed explanation for the 2004 Indonesian dengue epidemic. Infect Genet Evol. 2008;8: 191–204. doi: 10.1016/j.meegid.2007.12.005 18243816 10.1016/j.meegid.2007.12.005

[42] Huang J-H, Su C-L, Yang C-F, Liao T-L, Hsu T-C, Chang S-F, et al Molecular characterization and phylogenetic analysis of dengue viruses imported into Taiwan during 2008–2010. Am J Trop Med Hyg. 2012;87: 349–358. doi: 10.4269/ajtmh.2012.11-0666 22855770 10.4269/ajtmh.2012.11-0666 PMC3414576

[43] Kotaki T, Yamanaka A, Mulyatno KC, Labiqah A, Sucipto TH, Churrotin S, et al Phylogenetic analysis of dengue virus type 3 strains primarily isolated in 2013 from Surabaya, Indonesia. Jpn J Infect Dis. 2014;67: 227–229. 24858615 10.7883/yoken.67.227

