# Supplementary material for: Dengue in Bali: Clinical characteristics and genetic diversity of circulating dengue viruses
Source: PLoS Negl Trop Dis. 2017 May 22;11(5):e0005483. doi: 10.1371/journal.pntd.0005483 (PMC5456401; doi:10.1371/journal.pntd.0005483)
Supplement: S1 Checklist — (PDF) [file pntd.0005483.s001.pdf]

STROBE Statement—Checklist of items that should be included in reports of ***cross-sectional studies***

	Item No	Recommendation
**Title and abstract**	1	(*a*) Study design (cross-sectional) is described in the abstract
		(*b*) Method was described in the abstract such as the collection of demographic and clinical information, dengue screening using NS1 and IgM/IgG ELISAs, viral RNA extraction, serotyping using RT-PCR, and genotyping with sequencing. Findings were described such as the serotypes distribution and genotype grouping.
**Introduction**		
Background/rationale	2	Bali is hyperendemic for dengue. Most available reports on dengue virological and clinical features are from travelers' data. No data from local Balinese people available. We conducted study on dengue to understand the disease characteristics in local people.
Objectives	3	The objective of the study is to obtain comprehensive data on dengue disease in local Balinese. We conducted molecular surveillance to characterize clinical aspects and genetic diversity of the DENVs circulating in Bali.
**Methods**		
Study design	4	The study design is a cross-sectional prospective study, which was mentioned in the Materials and Method section.
Setting	5	The study setting, locations, and relevant dates, including periods of recruitment, exposure, follow-up, and data collection were described in Materials and Methods section.
Participants	6	Inpatients (above 14 years) presenting at the adult wards with fever >38°C accompanied by at least one sign of dengue such as malaise, arthralgia, rash, retro-orbital pain, DHF or DSS were enrolled in the study after providing written informed consents. We excluded patients with history of chronic illnesses, such as chronic liver disease, diabetes mellitus, chronic kidney disease, chronic lung disease, human immunodeficiency syndrome, and cardiac disease. Sera were collected during the acute phase (within the first five days of illness) and before discharge from the hospital.
Variables	7	The outcomes of this study are the serotypes/genotypes data, the diagnostics results, clinical and haematological data.
Data sources/ measurement	8*	N/A
Bias	9	Possible bias is because of inclusion of only adult patients was described in Discussion section.
Study size	10	The study size was obtained based on sample size calculation for cross-sectional study using the formula $N = \frac{Z\alpha^2 \times P \times Q}{d^2}$, with assumption the proportion of dengue infection in community P=4%, desired precision d=3%. A minimum of 164 participants was obtained from the calculation. We recruited 200 patients in this study.
Quantitative variables	11	N/A
Statistical methods	12	Statistical analysis was described in the method section.
**Results**		
Participants	13*	Numbers of individuals at each stage of study were described in the Results, including proportion of confirmed dengue.
		Exclusion of patient group based on its small sample size was described.

Descriptive data	14*	Characteristics of study participants (e.g. age and gender) were described.
Outcome data	15*	The proportion of DENV serotypes/genotypes and the clinical characteristics were described.
Main results	16	Main results were the serotype/genotype and phylogeny data of the DENV circulating in Bali.
		For clinical data, adjustment was performed for clinically relevant potential covariates, i.e. age, gender, recruitment site, infection status, and fever at day of presentation.
Other analyses	17	N/A
**Discussion**		
Key results	18	Key results were the serotype/genotype distribution and phylogenetic data. The clinical data were also presented.
Limitations	19	Study limitations were described in the study, including the inclusion of only adult patients, limited samples, and the duration of the study.
Interpretation	20	We confirmed the hyperendemicity of all four DENV serotypes where the circulating DENV included dominant local strains which were in circulation for several years and were related to recent imported dengue cases to other countries. Our study highlights Bali as a place with prominent genetic diversity of DENV and supports previous reports on its role in dengue transmission and mixing
Generalisability	21	N/A
**Other information**		
Funding	22	The study was funded by Warmadewa University and the Ministry of Research, Technology, and Higher Education of the Republic of Indonesia.

*Give information separately for exposed and unexposed groups.

Note: An Explanation and Elaboration article discusses each checklist item and gives methodological background and published examples of transparent reporting. The STROBE checklist is best used in conjunction with this article (freely available on the Web sites of PLoS Medicine at http://www.plosmedicine.org/, Annals of Internal Medicine at http://www.annals.org/, and Epidemiology at http://www.epidem.com/). Information on the STROBE Initiative is available at www.strobe-statement.org.
