# Supplementary material for: Dengue in Bali: Clinical characteristics and genetic diversity of circulating dengue viruses
Source: PLoS Negl Trop Dis. 2017 May 22;11(5):e0005483. doi: 10.1371/journal.pntd.0005483 (PMC5456401; doi:10.1371/journal.pntd.0005483)
Supplement: S1 Table — (PDF) [file pntd.0005483.s002.pdf]

Supplementary Table S1. Characteristics of sequenced samples.

No.	Sample ID	Serotype	Genotype	GenBank Accession No.	Length (nt)
1	WGY-039	DENV-1	I	KY006129	1485
2	WGY-055	DENV-1	I	KY006130	1485
3	WGY-066	DENV-1	I	KY006131	1485
4	SJN-001	DENV-1	I	KY006132	1485
5	SJN-015	DENV-1	I	KY006133	1485
6	SJN-016	DENV-1	I	KY006134	1485
7	SJN-018	DENV-1	I	KY006135	1485
8	SJN-049	DENV-1	I	KY006136	1485
9	SJN-050	DENV-1	I	KY006137	1485
10	SJN-051	DENV-1	I	KY006138	1485
11	WGY-004	DENV-2	Cosmopolitan	KY006139	1485
12	WGY-051	DENV-2	Cosmopolitan	KY006140	1485
13	WGY-082	DENV-2	Cosmopolitan	KY006141	1485
14	SJN-006	DENV-2	Cosmopolitan	KY006142	1485
15	SJN-020	DENV-2	Cosmopolitan	KY006143	1485
16	WGY-014	DENV-3	I	KY006144	1479
17	WGY-020	DENV-3	I	KY006145	1479
18	WGY-022	DENV-3	I	KY006146	1479
19	WGY-023	DENV-3	I	KY006147	1479
20	WGY-027	DENV-3	I	KY006148	1479
21	WGY-031	DENV-3	I	KY006149	1479
22	WGY-032	DENV-3	I	KY006150	1479
23	WGY-086	DENV-3	I	KY006151	1479
24	SJN-003	DENV-3	I	KY006152	1479
25	SJN-005	DENV-3	I	KY006153	1479
26	WGY-009	DENV-4	II	KY006154	1485
27	WGY-011	DENV-4	II	KY006155	1485
28	WGY-080	DENV-4	II	KY006156	1485
