# Supplementary material for: Dengue in Bali: Clinical characteristics and genetic diversity of circulating dengue viruses
Source: PLoS Negl Trop Dis. 2017 May 22;11(5):e0005483. doi: 10.1371/journal.pntd.0005483 (PMC5456401; doi:10.1371/journal.pntd.0005483)
Supplement: S2 Table — (PDF) [file pntd.0005483.s003.pdf]

Supplementary Table S2. Clinical parameters of dengue patients in relation to the disease severity.

Variables	DF (N=75)		DHF (N=68)	
	**N (%)**	**RR (95% CI)**	**N (%)**	**RR (95% CI)**
Malaise	70 (93.3)	1.40 (0.49 - 3.95)	63 (92.6)	0.63 (0.20 -1.93)
Nausea	66 (88.0)	1.34 (0.57 - 3.13)	59 (86.8)	0.66 (0.26- 1.64)
Loss of appetite	61 (81.3)	0.96 (0.47 - 1.99)	58 (85.3)	1.05 (0.44 - 2.28)
Headache	52 (69.3)	0.78 (0.41 - 1.46)	54 (79.4)	1.38 (0.67 - 2.84)
Myalgia	44 (58.7)	0.84 (0.46 - 1.55)	45 (66.2)	1.37 (0.68 - 2.76)
Vomiting	38 (50.7)	0.80 (0.48 - 1.31)	42 (61.8)	1.35 (0.74 - 2.46)
Arthralgia	38 (50.7)	1.17 (0.63 - 2.19)	38 (55.9)	0.85 (0.45 - 1.62)
Abdominal pain	19 (25.3)	0.73 (0.40 - 1.35)	24 (35.3)	1.30 (0.72 - 2.34)
Retro-orbital pain	17 (22.7)	0.98 (0.51 - 1.89)	19 (27.9)	0.93 (0.48 - 1.81)
Bleeding	11 (14.7)	0.68 (0.35 - 1.32)	19 (27.9)	1.41 (0.80 - 2.49)

Relative risk (RR) and confidence intervals (CI) were calculated based on modified Poisson regression adjusted for age, gender, primary/secondary infection, recruitment site, and fever day at presentation.
